# Prevalence of HIV/AIDS Infection among Sexually Active Women in Ethiopia: Further Analysis of 2016 EDHS

**DOI:** 10.1155/2022/8971654

**Published:** 2022-11-07

**Authors:** Solomon Sisay Mulugeta, Selamawit Getachew Wassihun

**Affiliations:** ^1^Department of Statistics, Debre Tabor University, Amhara, Debre Tabor, Ethiopia; ^2^Department of Statistics, Mekdela Amba University, Amhara, Ethiopia

## Abstract

**Background:**

The issue of HIV/AIDS is prevalent around the world and in Ethiopia as well. The aim of this study was to determine the prevalence and risk factors of HIV/AIDS infection among sexually active women in Ethiopia.

**Methods:**

For this study, data were obtained from the Ethiopia Demographic and Health Survey conducted in 2016. This study contains 11,729 women who have had at least one episode of sexual intercourse in their lifetime. Interviewers for voluntary HIV testing collected finger-prick blood specimens from women who agreed to be tested for HIV. Based on factors at the individual and community levels, a multilevel logistic regression model was used.

**Results:**

The study found that 2% of 11,729 sexually active women tested positive for HIV from all regions who received voluntary counseling and testing. The intraclass correlation coefficient findings showed that 32.844% of HIV/AIDS transmission among sexually active women was the result of community-level factors. Variables at the individual level were women of age 16–24 (AOR = 0.18; 95% CI: 0.11–0.29), women of age 25–34 (AOR = 0.733; 95% CI: 0.55–0.98), women with primary education level (AOR = 1.8; 95% CI: 1.23–2.57), more than one sexual accomplice (AOR = 1.33; 95% CI: 0.613–2.87), and women's age at first sexual intercourse between 25 and 34 (AOR = 0.57, 95% CI: 0.301, 1.06); these were the most significant determinants of HIV/AIDS infection. According to community-level factors, there was a lower HIV prevalence rate among rural women (AOR = 0.22; 95 percent CI: 0.13–0.36), and women in the Gambela region (AOR = 4.1; 95 percent CI: 1.99–8.34) also had higher HIV prevalence rates.

**Conclusions:**

The prevalence of HIV infection among sexually active women varies by region, with urban women more likely to contract the virus. Women who had more than one regular sexual partner and had their first sexual encounter at a younger age are at an increased risk of contracting HIV/AIDS. According to the study, the government should focus more support on high-risk clusters, mainly in urban areas, as well as on regions with high rates of HIV/AIDS infection.

## 1. Background

Globally, over seventy-six million humans have been abscessed with the human immunodeficiency virus (HIV), and HIV infection has triggered more than 35 million deaths since its inception [1]. HIV/AIDS is a global community health matter. In 2018, approximately 37.9 million people were living with HIV worldwide, with 2.1 million newly diagnosed. The sub-Saharan region is the most affected in the world, with 25.6 million HIV-positive people [2, 3].

HIV/AIDS is a major public health concern and a leading cause of death in many African countries. Despite accounting for about 15.2% of the world's population, Africans accounted for more than two-thirds of the total infected worldwide, with 35 million infected, 15 million of who have already died [4]. The youth prevalence of HIV was twice as high among women (15–24 years) in the region in 2016 (3.4% vs. 1.6%), and differences between sexes are more evident in certain countries. According to reports conducted in the district, young women are more likely to face abuse from their spouses than women of any age [5]. In addition, this increases the chances of HIV transmission; for instance, a South African study found that younger women who had experienced personal savagery were half as likely to have HIV as those who had not [6].

In sub-Saharan Africa, Ethiopia has the highest number of people living with HIV/AIDS. It is an essential part of emerging nations' health systems [7, 8]. HIV is prevalent in Ethiopia, but the pattern of transmission differed from country to country. This could be because of an absence of antiretroviral therapy (ART) drug inclusion and adhesiveness, which has brought about a high death rate among HIV-positive individuals [1].

In Ethiopia, the general pattern of the pervasiveness rate fluctuates from one year to another and from one locale to another. A pattern investigation of the country from 1982 to 2011 shows a consistent progressive ascent in HIV/AIDS pervasiveness until the last part of the 1990s, trailed by a consistent downfall after 2000 [9]. The public grown-up HIV pervasiveness rate was assessed to be 0.2% in 1985, 3.2% in 1995, and 1.4% in 2005 [10]. Nonetheless, as per the 2011 EDHS report, the commonness rate expanded simply somewhat in 2011 compared with 2005. The epidemic remains highly heterogeneous by region, with the lowest (0.9%) in SNNPR and the highest (6.5%) in Gambela [11, 12]. The survey also reveals a gender disparity (1.9% for adult women versus (1%) for adult men. As per this, the commonness pace of women and men in the age bunch 15–49 was 1.5%. HIV prevalence is also becoming more concentrated in large urban areas and along major transportation corridors [12].

Ethiopia has gained ground in lessening the quantity of HIV/AIDS cases in the nation, yet the changes observed are deficient in contrast with the ideal objectives of the reaction to the pestilence [13, 14]. Given the size of the populace and the greatness of the harm incurred, it will require a long while to see critical decreases in HIV commonness and frequency with purposeful and supported endeavors, keeping in mind that there have been progresses in the accessibility, openness, and use of HIV/AIDS anticipation, care, backing, and treatment administrations, as well as upgrades in the scourge of the executives and expanding asset accessibility, we need more endeavors to control the plague [1, 15–17]. Subsequently, the motivation for this study was to investigate the role of socioeconomic, HIV/AIDS mindfulness, and sexual conduct factors in understanding the prevalence and risk factors for HIV disease among Ethiopian sexually active women.

## 2. Methods

### 2.1. Study Population

The study used the nationally set of representative cross-sectional data extracted from the EDHS 2016. The Central Statistical Agency (CSA) conducted the 2016 Ethiopia Demographic and Health Survey (2016 EDHS) from January 18, 2016, to June 27, 2016. Ethiopia's demographic and health survey 2016 select a total of 18,008 households for the sample, of which 17,067 were occupied. Of the occupied, 16,650 were successfully interviewed, yielding a response rate of 98%. The total household size was 16,650, and of these, 15,683 were eligible women. In the sample of interviewed women, 15,683 responded correctly [12]. Our study includes 11,729 women who have had at least one sexual intercourse in their lifetime as well as voluntary HIV testing. This study does not include women who have never had a sexual relationship. The samples were selected using a two-stage stratified cluster sampling technique. Each region was stratified into urban and rural areas, yielding 21 sampling strata. Samples of EAs were selected independently in each stratum in two stages. In the first stage, a total of 645 EAs (202 in urban areas and 443 in rural areas) were selected with a probability proportional to each EA's size. In the selection of the second stage, a fixed number of 28 households per cluster were selected with an equal probability selection from the newly created household listing. All women who have an age group between 15 and 49 and who were either permanent residents of the selected households or visitors who stayed in the household at night before the survey were eligible to be interviewed [12].

### 2.2. Measurement of Variables

#### 2.2.1. Response Variables

The dependent variable is dichotomous “HIV status of the women in Ethiopia” (HIV positive = 1; HIV negative = 0).

### 2.3. Explanatory Variables

#### 2.3.1. Individual Level Variables

Individual-level covariates associated with HIV status in Ethiopian women were classified as the women's background, socioeconomic status, and demographic characteristics: age, education, sex, and wealth index; HIV/AIDS awareness, which includes general knowledge, transmission modality, and methods of infection prevention. As a result, it can be divided into two categories: no and yes. In addition, marital union status, age at first sex, and multiple sexual partners are all factors in sexual behavior [12].

### 2.4. Factors at the Community Level

Region and urban rural residence were considered community-level factors incur directly from the EDHS.

### 2.5. Management and Analysis of Data

In this study, data were extracted and decoded using SPSS software version 23, and the decoded data were analyzed using STATA version 14. To characterize the study participants, descriptive statistics such as frequencies and percentages were used. A multilevel study design does not consider individual women to be independent of one another. In this study, patients are nested by cluster (enumeration areas). In this case, the conventional regression model is meaningless. Because of this, a multilevel logistic regression model was developed to determine the prevalence of the HIV/AIDS pandemic among women who are sexually active.

The null model (model I) is the first of four successive multilevel model analyses, and it is equipped with no explanatory variable at the individual and community levels to recognize the presence of a potential contextual result. The 2^nd^ model (model I) was set by including all individual-level variables. This step evaluates the significance of each individual-level explanatory variable, the significance of each predictor, and changes in the first- and second-level variance terms. By incorporating all community-level variables, the third model (Model II) was developed. Individual-level and community-level factors were included in the 4^th^ model (model III) [18, 19].

### 2.6. Multilevel Logistic Regression Analysis

Because of the data's hierarchical nature, multilevel logistic regression fashions were used to determine factors related to HIV/AIDS status among women [18]. A two-level multilevel model was used to model the log of the likelihood of HIV/AIDS status as described in the following equation:

(1) $$\begin{array}{c} \text{Log}\left[\frac{\pi_{ij}}{1-\pi_{ij}}\right]=\beta_{0}+\beta_{1}X_{ij}+\beta Z_{ij}+u_{j}+e_{ij}, \end{array}$$

where *i* and *j* are the level 1 and level 2 units, respectively; *X* mention to individual-level factors and *Z* mention to the community-level variable; *π*_*ij*_ denotes to the probability of HIV/AIDS status for the *i*^th^ women in the *j*^th^ community; and the *β* bespeak the fixed coefficients. In the absence of predictor influence, the intercept-the effect on the probability of HIV/AIDS status among sexually active women is mentioned by *β*_0_; *u*_*j*_ demonstrate the random effect (the issue of the community on HIV status for the *j*^th^ community, then *e*_*ij*_ also demonstrate the random errors at the individual level. Deviance information criteria (DIC), Akaike's information criterion (AIC), and Bayesian's information criterion (BIC) were used to compare models [20]. As the final model of the analysis, the lowest information criterion value was chosen. Odds ratios (ORs) with 95% confidence intervals (CIs) were used to determine statistical significance. *P*-value ≤0.05 is well thought out statistically significant. To measure the degree of variation between enumeration areas, the measures of variation were summarized using the ICC, MOR, and PCV. The following formula was used to compute the ICC: *ICC*=*V*_*A*_/*V*_*A*_+(*π*^2^/3)=*V*_*A*_/*V*_*A*_+3.29, where, in each model described elsewhere, *V*_*A*_ represents the variance estimation. The total variation attributed to individual or community-level factors was calculated using PCV at each model: PCV=(*V*_*A*_ − *V*_*B*_)/*V*_*A*_, where *V*_*A*_ is the initial model's variance and *V*_*B*_*s* the model's variance with more terms [21]. The MOR compares two women from two different randomly selected communities and measures unexplained cluster heterogeneity as well as variation between clusters by comparing two women from two different randomly selected communities. The following formula was used to compute it: $\mathrm{MOR}=\exp\left(\sqrt{2\times V_{A}\times 0.6745}\right)\approx \exp\left(0.95\sqrt{V_{A}}\right)$, where *V*_*A*_ represents the variance at the community level, and MOR is always≥ 1. There is no difference between clusters if the MOR is 1 [19, 20].

## 3. Results

### 3.1. Sociodemographic Characteristics of Sexually Active Women

A total of 11,729 sexually active women received VCT offerings from all regions covered in this study.  Table 1 depicts the distribution of seropositive people based on a few demographic/HIV/AIDS-related risk behavior variables. Sero-positivity (HIV infected) is higher in urban women (4.6%) than in rural women (0.8%). The majority of HIV-positive women were found in Gambela (5.1%), Addis Ababa (4.7%), Dire Dawa (2.9%), and Harari (2.9%). According to the age of women, the prevalence of HIV positivity among women aged 25 and up was high (25–34 (2.3%) and over 34 (%)). In terms of age at first sex, the results show that sexually active women over the age of 35 were highly affected by the virus (2.9%), with the age group 18–24 (2.2%) revealing the highest proportion of HIV positives. In this study, among the HIV-positive respondents most of them come from wealthy (3.4%) families, and the majority of them are educated (primary (2.8%) and secondary and above (3%)). The majority of HIV-positive respondents in this study were Christians (2.9%), and they had never married (3.5%). Except for religion (*x*^2^-value = 3.4, *P*-value = 0.337), there is a significant association between all sociodemographic characteristics (Table 1).

### 3.2. Women's HIV/AIDS Status: A Multilevel Logistic Regression Analysis

Table 2 summarizes the results of the multivariable multilevel binary logistic regression model for both individual and community-level variables. The model comparison result revealed that model III is a better fit for the data than other reduced models because it has the smallest AIC, BIC, and deviance statistics (Table 3).

### 3.3. Individual Level Variables

The variable age of women between 16 and 24 (AOR = 0.18; 95% CI: 0.11–0.29), age between 25 and 34 (AOR = 0.733; 95% CI: 0.55–0.98), women with primary education (AOR = 1.8; 95% CI: 1.23–2.57), more than one sexual accomplice preceding to the survey (AOR = 1.33; 95% CI: 0.613–2.87), and women's age at first sexual intercourse between 25 and 34 (AOR = 0.57; 95% CI: 0.301, 1.06) were the most significant (Table 2).

### 3.4. Community-Level Factors

Women in rural areas were less likely to be HIV positive (AOR = 0.22; 95% CI: 0.13–0.36) than urban women. Women found in the Somalia region (AOR = 0.17; 95% CI: 0.036–0.85) and Gambela region (AOR = 4.1; 95% CI: 1.99–8.34) were the most significant determinant factors of HIV/AIDS infection among women who are sexually active (Table 2).

### 3.5. Random Effect Measures of Variation

The prevalence of the HIV/AIDS pandemic among sexually active women varied across communities (Table 3). According to the ICC, community-level factors accounted for 32.844% of the HIV/AIDS infection rate among sexually active women. According to the PVC findings, the full-model explained approximately 67.1% of the HIV/AIDS infections in clusters. Furthermore, the MOR confirmed that the HIV/AIDS infection was caused by community-level factors. In the null model, the MOR for the HIV/AIDS infection among sexually active women was 3.34, indicating that there was variation across communities. In the model III, the unexplained community variation in the HIV/AIDS infection among sexually active women was reduced to a MOR of 1.99. The unexplained community variation in HIV/AIDS infection among sexually active women was reduced to a MOR of 1.99 in model III. This meant that the effects of clustering remained statistically significant in the full-models even after controlling for all other variables (Table 3).

## 4. Discussion

Using data from the Ethiopian Demographic and Health Survey 2016 [12], the purpose of this study was to identify demographic, socioeconomic, HIV/AIDS awareness, and sexual behavior factors associated with HIV/AIDS infection among sexually active women.

Women's residence had a significant impact on the prevalence of HIV/AIDS infection. This finding is consistent with the findings of an Ethiopian study [1, 11, 22] and sub-Saharan Africa [23, 24]. The proportion of respondents who lived in cities was higher than the proportion of respondents who lived in rural areas. The prevalence of high-risk HIV infection (HIV seropositive) and transmission practices among urban women was high. Most urban areas of Ethiopia are characterized by risky populations such as long-distance drivers, sex workers, and military personnel who gather in places with bars, pensions, guest houses, hotels, massage houses, shisha and khat houses, night clubs, drinking establishments, and tourist-friendly settings [1]. Furthermore, the population density was high in urban and increased AIDS knowledge was linked to more frequent high-risk sexual practices [25]. In terms of regional differences, the likelihood of HIV infection among women who are sexually active was higher in the Gambela region and lower in the Somalia region when compared to the Tigray region. The founding showed that the odds of HIV cases among sexually active women in Gambela regions were 4.1 times higher than in Tigray regions. There are 0.17 times as many HIV-positive cases in Somalia as in Tigray. A study conducted in Ethiopia confirmed this finding [1, 11, 22, 26, 27]. The prevalence of HIV infection varies across Ethiopia's eleven regions. Women face financial, societal, and political discrimination in the community, which may alter their HIV/AIDS disclosure. Many of them are also victims of various forms of violence, ranging from sexual conflict to degrading traditional practices that boost their risk of HIV infection.

HIV infection is more common in educated women than in women with no or only preschool education. HIV infection is 1.8 times more likely in sexually active women with primary level education than in women with no education or preschool level education. This means that sexually active women with primary school education are more likely to be abscessed with HIV than those with no education or only preschool education. This study was coinciding with other studies [11, 22, 28, 29]. A higher level of education is linked to an increased risk of HIV infection because the more educated were wealthier, more mobile, and have more sexual partner networks.

In sexually active women between 16–24 and 25–34 years, the probability of HIV infection was 0.8 and 0.733 times lower than that in women under 15 years old. This result is similar to a previous study, which found that the disease is especially prevalent in young women and adolescent girls between 15 and 24 years old [27, 30]. This creates a spontaneous sense, as the risk of being HIV positive increases with age, as does the risk of transmitting it unless people are on treatment and virally suppressed. Women who had more than one regular sexual partner had a higher risk of being HIV positive. Women who had more than one sexual partner were 1.33 times more likely to be HIV positive. The study discovered that a younger age at first sexual intercourse and a higher frequency of new sexual partners are related to an increased risk of HIV infection in women. Multiple sexual partners are independently linked to an increased risk of HIV infection [31–33].

### 4.1. Strength and Limitations of the Study

This study uses a nationally representative survey dataset, which enhances inferences for the entire country level. The major strength was that interaction effects were examined in the study. In the survey, participants were asked to recall events from five years ago, and some details may have been forgotten. The cross-sectional nature of the data also prevents causal relationships between outcome and exposure variables from being identified. Due to these limitations, estimates for specific countries may change over time as a result of the self-reported data and should be considered cautiously since they are based on self-reported values from a nationally representative survey. As a national representative dataset, the EDHS is designed and deployed rigorously by the Centers for Disease Control and Prevention. Despite these limitations, this allows the results to be generalized across the country. It will also be possible to compare the results internationally due to the use of similar instruments across countries.

## 5. Conclusion

In the path of HIV prevention, treatment, care, and backing administrations, HIV testing is essential. This research was undertaken to identify demographic, socioeconomic, HIV/AIDS awareness, and sexual behavior factors related to HIV/AIDS among women who are sexually active. The chances of contracting HIV increased for women in the country's urban communities, regardless of how the prevalence of the disease varies by region. Women with more schooling are more likely to contract HIV than women without any instruction or just a preschool education. HIV disease is more likely to occur in women who have had more than one sexual partner and who had their first sexual experience at a younger age. The findings of this study suggest that the government should provide more assistance and focus on regions with high rates of HIV/AIDS infection. Furthermore, prevention efforts should concentrate on gatherings with high risks, especially in urban areas and among women with a low level of education.

## Figures and Tables

**Table 1:** Association between sociodemographic characteristics and HIV/AIDS status of women in Ethiopia, 2016 EDHS.

Characteristics	HIV-positive women		Statistical comparison	
	N(239)	% [2]	*x* ^2^-value	*P* value
*Region*				
Tigray	18	1.4	133.33	≤0.0001
Afar	16	1.8	Df = 10	
Amhara	21	1.5		
Oromia	14	1		
Somalia	2	0.2		
Benishangul	14	1.5		
SNNPR	10	0.7		
Gambela	43	5.1		
Harari	16	2.9		
Addis Ababa	63	4.7		
Dire Dawa	22	2.9		
*Place of residence*				
Urban	172	4.6	178.713	≤0.0001
Rural	67	0.8	Df = 1	
*Age of women*				
≤15	0	0	28.71	≤0.0001
16–24	20	0.8	Df = 3	
25–34	111	2.3		
≥35	108	2.6		
*Economic status*				
Poorer	37	0.8	99.202	≤0.0001
Middle	12	0.8	Df = 2	
Richer	190	3.4		
*Marital status*				
Never married	59	3.5	23.037	≤0.0001
Married	163	1.7	Df = 2	
Separated	17	2.6		
*Education level*				
None	56	1	51.098	≤0.0001
Primary	109	2.8	Df = 2	
Secondary and above	74	3		
*Sexual partner*				
Only one	10	1.7	31.9	≤0.0001
More than one sexual accomplice in the preceding to the survey	229	2.1	1	
*Age at first sex*				
<18	54	2.1	51.38	≤0.0001
18–24	160	2.2	Df = 3	
25–34	24	1.5		
≥35	1	2.9		
*Religion*				
Christians	150	2.9	3.4	0.337
Muslim	37	0.8	Df = 2	
Other	52	2.4		
*Ever heard about HIV/AIDS*				
No	2	0.5	4.74	≤0.0001
Yes	237	2.1	Df = 1	

**Table 2:** Multilevel multivariable logistic regression analysis of both individual and community-level factors with HIV/AIDS status of women in Ethiopia, 2016 EDHS.

Individual and community-level variables	Models			
	Null model AOR (95% CI)	Model I AOR (95% CI)	Model II AOR (95% CI)	Model III AOR (95% CI)
*Region*				
Tigray	Ref		Ref	Ref
Afar			1.3 (0.56, 2.94)	1.6 (0.683, 3.53)
Amhara			1.3 (0.6, 2.70)	1.3 (0.56, 2.71)
Oromia			0.9 (0.4, 2.03)	0.83 (0.37, 1.9)
Somalia			0.13 (0.03, 0.591)^*∗∗*^	0.17 (0.036, 0.85)^*∗*^
Benishangul			1.31 (0.6, 3.03)	1.46 (0.64, 3.34)
SNNPR			0.75 (0.31, 1.81)	0.72 (0.3, 1.74)
Gambela			3.43 (1.71, 6.91)^*∗∗*^	4.1 (1.99, 8.34)^*∗∗∗*^
Harari			1.15 (0.5, 2.62)	1.3 (0.56, 2.85)
Addis Ababa			1.24 (0.63, 2.5)	1.11 (0.56, 2.17)
Dire Dawa			0.995 (0.46, 2.18)	0.92 (0.42, 1.995)
*Place of residence*				
Urban	Ref		Ref	Ref
Rural			0.172 (0.11, 0.26)^*∗∗∗*^	0.22 (0.13, 0.36)^*∗∗∗*^
*Age of women*				
≤15	Ref		Ref	Ref
16–24	0.17 (0.09, 0.28)^*∗∗∗*^			0.18 (0.11, 0 .29)^*∗∗∗*^
25–34	0.75 (0.56, 0.996)^*∗*^			0.733 (0.55, 0.98)^*∗∗*^
≥35	0.41 (0.26, 0.63)			0.91 (0.82, 1.02)
*Economic status*				
Poorer	Ref		Ref	Ref
Middle	0.91 (0.46, 1.8)			0.95 (0 .48, 1.9)
Richer	3.1 (2.03, 4.73)^*∗∗∗*^			1.41 (0.833, 2.4)
*Marital status*				
Never married	Ref		Ref	Ref
Married	0.6 (0.39, 0.89)^*∗*^			0.74 (0.5, 1.13)
Separated	0.92 (0.45, 1.9)			1.5 (0.73, 2.96)
*Education level*				
None	Ref		Ref	Ref
Primary	2.33 (1.63, 3.3)^*∗∗∗*^			1.8 (1.23, 2.57)^*∗∗*^
Secondary and above	1.81 (1.195, 2.74)^*∗∗*^			1.1 (0.718, 1.68)
*Number of sexual partner*				
Only one	Ref		Ref	Ref
More than one sexual accomplice in the preceding to the survey	1.2 (0.53, 2.46)^*∗*^			1.33 (0.613, 2.87)^*∗*^
*Age at first sex*				
<18	Ref		Ref	Ref
18–24	0.7 (0.44, 0.993)^*∗*^			0.84 (0.55, 1.3)
25–34	0.37 (0.199, 0.68)^*∗∗*^			0.57 (0.301, 1.06)^*∗*^
≥35	1.85 (0.19, 18.11)			4.93 (0.51, 47.94
*Ever heard about HIV/AIDS*				
No	Ref		Ref	Ref
Yes	2.86 (0.61, 13.4)			4.13 (0.83, 20.6)

^*∗*^significant at *P* value <0.05. ^*∗∗*^significant at *P* value <0.01. ^*∗∗∗*^significant at *P* value <0.001.

**Table 3:** Measure of variation on individual and community-level factors associated with HIV/AIDS status of women in Ethiopia, 2016 EDHS.

Measure of variation	Models			
	Null model	Model I	Model II	Model III
Community variance (SE)	1.61 (0.333)	0.814 (0.215)	0.64 (0.191)	0.53 (0.18)
ICC (%)	32.844	19.84	16.32	13.77
PVC (%)	1	49.44	60.25	67.08
MOR	3.34	2.36	2.14	1.99
*Model fit statistics*				
−2^*∗*^LL (DIC)	2252.394	2104.53	2096.604	2012.554
AIC	2256.394	2134.53	2122.609	2064.553
BIC	2271.134	2245.073	2218.416	2256.164

## Data Availability

In this study, the Ethiopia Demographic Health Survey data were used. These data can be requested from the Demographic Health Survey website, https://www.dhsprogram.com.

## Abbreviation

AIDS: Acquired immune deficiency syndrome
CSA: Central Statistical Agency
DHS: Demographic and Health Survey
EDHS: Ethiopia Demographic and Health Survey
HIV: Human immunedeficiency virus
ICC: Intraclass correlation coefficient
LR: Likelihood-ratio
MOR: Median odds ratio
PCV: Proportional change in variance
SNNPR: South Nations, Nationalities, and People Regional State
VCT: Voluntary counseling and testing.
